# New-Onset Ocular Myasthenia Gravis After Booster Dose of COVID-19 Vaccine

**DOI:** 10.7759/cureus.27213

**Published:** 2022-07-24

**Authors:** Ana Abicic, Barbara Sitas, Ivan Adamec, Ervina Bilic, Mario Habek

**Affiliations:** 1 Neurology, General Hospital Zabok, Zabok, HRV; 2 Department of Neurology, University Hospital Centre Zagreb, Zagreb, HRV; 3 Neurology, School of Medicine, University of Zagreb, Zagreb, HRV

**Keywords:** diplopia, bnt162 vaccine, autoimmune diseases, covid-19 vaccines, myasthenia gravis

## Abstract

Coronavirus disease 2019 (COVID-19) vaccines have been reported as possible triggers of the production of antibodies pathogenic to the peripheral nerve and neuromuscular junction. We report on a patient who experienced vertical diplopia three weeks after the booster dose of the Pfizer-BioNTech vaccine (Comirnaty®). The diagnosis of myasthenia gravis (MG) was established based on highly positive antibodies to the nicotinic acetylcholine receptor (nAChR). Treatment with pyridostigmine and prednisone was started with gradually raising doses. On a follow-up exam two months after treatment initiation, clinical improvement was noted with an almost normal bulbomotor examination. The occurrence of diplopia following COVID-19 vaccination should raise suspicion of new-onset ocular MG and testing for anti-nAChR antibodies is advised.

## Introduction

Ocular myasthenia gravis (MG) is an autoimmune disorder characterized by weakness of ocular muscles, which clinically presents with isolated ptosis, diplopia, or both [1]. Positive antibodies to the nicotinic acetylcholine receptor (nAChR) are the most specific finding for the diagnosis, although, in approximately half of the patients with ocular MG, the titer will be negative [1]. Coronavirus disease 2019 (COVID-19) vaccines have been reported as possible triggers for the production of antibodies pathogenic to the peripheral nerve and neuromuscular junction [2-5]. However, MG following COVID-19 vaccination is rarely reported [3-5]. We report on a case of new-onset myasthenia with ocular presentation occurring shortly after a booster dose of the COVID-19 vaccine.

## Case presentation

**Figure 1:**
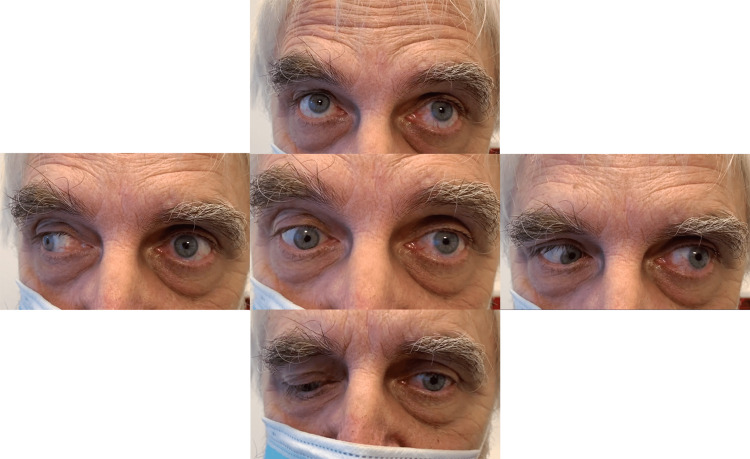
Initial bulbomotor examination revealed skew deviation with the right eye in depression. Left eye movement was paretic for all directions, most pronounced for horizontal movements.

A 65-year-old man presented to the emergency department with vertical diplopia. He had woken up the day before with double vision. Diplopia was present continuously regardless of the time of day, with no signs or symptoms of other muscle weakness. His past medical history was unremarkable, and he did not take any medications. Three weeks before developing diplopia the patient received the booster dose of the Pfizer-BioNTech vaccine (Comirnaty®). Six months prior, the patient was vaccinated with two doses of the ChAdOx1 nCoV-19 vaccine (Vaxzevria®) with no significant side effects. Neurologic examination in the emergency department revealed skew deviation with the right eye in depression. Pupils were equal in size with normal light reactions. Left eye movement was paretic for all directions, most pronounced for horizontal movements, as shown in Figure 1.

**Figure 2:**
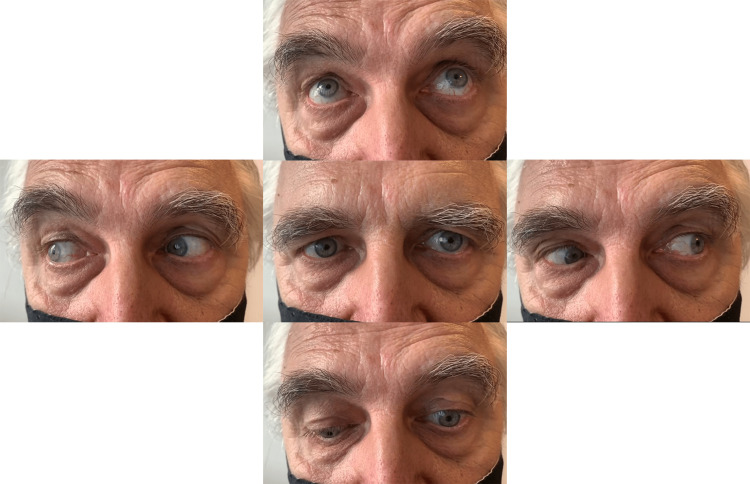
Bulbomotor examination two months after treatment initiation showed significant improvement, left eye remained paretic only when looking down.

The patient reported double vision in all directions, except when looking up and straight. The rest of the neurological examination was normal, including the tests of fatigability. Computed tomography (CT) scan of the brain was unremarkable. Routine laboratory examination including complete blood count, blood glucose, creatinine, blood urea nitrogen, sodium, potassium, C-reactive protein, prothrombin time, and activated partial thromboplastin time were normal. Brain magnetic resonance imaging (MRI) demonstrated small nonspecific subcortical white matter T2 hyperintense lesions in both cerebral hemispheres. MRI angiography showed no signs of aneurysm. Glycated hemoglobin and thyroid hormones were normal. Intramuscular prostigmin test was negative. Because of the presumed autoimmune etiology, intravenous methylprednisolone was administered, 1000 mg daily for five days, however with no improvement. Subsequently, immunology tests revealed highly positive anti-nAChR antibodies (>8,00 nmol/L; reference values: <0.5 negative, 0.51-0.99 borderline positive, 1.00-1.99 positive, >2.00 highly positive). Antibodies to muscle-specific tyrosine kinase (MuSK), antinuclear antibodies, rheumatoid factor, and antiganglioside antibodies were all negative. Chest CT scan showed no sign of thymoma. Repetitive nerve stimulation test was negative. As the results of the diagnostic workup were consistent with MG, the patient was started on 180 mg of pyridostigmine daily, which he tolerated well. In the second week, the dose of pyridostigmine was raised to 300 mg daily and 10 mg of prednisone daily was added. In the following weeks, the dose of prednisone was gradually raised to 20 mg daily. On a follow-up exam two months after the treatment initiation, significant improvement in the bulbomotor examination was noted (Figure 2). The patient reported vertical diplopia only in the far left and down eye position.

## Discussion

We report on a patient who experienced new-onset MG after the booster dose of the Pfizer-BioNTech vaccine. Previous studies have suggested that infection with severe acute respiratory syndrome coronavirus 2 (SARS-CoV-2) may cause dysregulation of the cholinergic system [6]. Although it is not fully understood how the infection contributes to the development of MG, loss of immunological self-tolerance following COVID-19 may unmask or trigger latent myasthenia [7]. A potential antigenic similarity and interaction between the SARS-CoV-2 proteins and nAChR at the neuromuscular junction might trigger the autoimmune response [7]. There have been rare previous reports of new-onset myasthenia after the COVID-19 vaccination. COVID-19 vaccines introduce the spike (S) protein of the SARS-CoV-2 virus into the organism leading to protective antibody production [8]. The S protein has a sequence similar to neurotoxins that can bind to nAChR [6]. This may explain why antibodies to the SARS-CoV-2 spike could in fact lead to dysfunction of the neuromuscular junction.

A case of new-onset ocular MG after the first dose of the ChAdOx1 nCoV-19 vaccine has been previously described [3]. In that case, the patient had a positive titer of anti-nAChR antibodies in the serum, the same as in the current report, and a pre-existing memory cell population was postulated because of disease onset within only a few days from vaccination [3]. Another plausible explanation of new MG occurrence following vaccination is bystander activation, meaning that vaccine response causes the release of previously sequestered self-antigens, resulting in activation of autoreactive T-cells [9]. Two other cases have reported new-onset MG following two doses of the Pfizer-BioNTech vaccine [4,5]. The current case is distinct from those previously published as our patient showed no signs or symptoms of MG after vaccination with the ChAdOx1 nCoV-19 vaccine. However, the patient developed MG three weeks after the booster dose with the Pfizer-BioNTech vaccine. One possible explanation might be the fact that vaccination with the Pfizer-BioNTech vaccine leads to higher antibody production compared to the ChAdOx1 nCoV-19 vaccine, rendering it more immunogenic [8]. This in turn may have led to stimulation of the innate immune system and development of bystander activation.

A study by Watad et al. [10] evaluated immune-mediated disease flares or new disease onset in 27 subjects within 28 days of SARS-CoV-2 vaccination in five large tertiary centers in countries with early vaccination adoption. In that cohort, two cases of new-onset MG were reported, both after receiving the second dose of the Pfizer-BioNTech vaccine [10]. Based on the level of population exposure in the regions covered by those centers, the authors concluded that incidence of the autoimmune conditions associated with COVID-19 vaccines appears to be rare [10].

Studies of SARS-CoV-2 vaccination in patients with established diagnosis of MG suggest that it might rarely lead to disease worsening [11,12]. In a retrospective study on an Italian cohort of 80 patients, transient worsening after vaccination was noted in 5% of patients [11]. In a prospective study following 343 Japanese patients, transient worsening was observed in 1% of patients and the response to treatment was favourable [12]. Therefore, the authors concluded that vaccination should be recommended for patients diagnosed with MG [11,12].

## Conclusions

In the present case, we describe a patient who developed diplopia following the Pfizer-BioNTech vaccine. The workup led to the diagnosis of MG and significant improvement was noted on pyridostigmine and corticosteroid treatment. This case illustrates that the occurrence of diplopia following COVID-19 vaccination should raise suspicion of new-onset ocular MG and testing for anti-nAChR antibodies is advised. 

Although COVID-19 vaccines may be associated with new onset of autoimmune diseases, the risk of COVID-19 vaccination-related complications is still low compared to the serious adverse events and neurological complications of COVID-19 and vaccination should be advocated. Further studies are warranted to understand better the immune response to COVID-19 vaccines and their impact on autoimmune disease flares.
